# Highly efficient photocatalytic degradation of dyes and anticancer activity of eco-friendly synthesized Ag/Ag_2_O/γ-Fe_2_O_3_ nanoparticles using *Cistus monspeliensis* leaf extract

**DOI:** 10.1039/d5ra08684a

**Published:** 2026-01-28

**Authors:** Radja Nada Boucetta, Malika Khelfaoui, Nesrine Ammouchi, Sabrine Boucetta, Mohamed Nadir Khelifi, Erkan Can, Atmane Djermoune, Farid Ait Merzeg, Widad Sobhi, Faycal Djazi

**Affiliations:** a Department of Process Engineering, Faculty of Technology, 20 August 1955 University El-Hadaeik Road, P.O. Box 26 Skikda 21000 Algeria rn.boucetta@univ-skikda.dz; b Laboratoire de Recherche sur la Physico-Chimie des Surfaces et Interfaces (LRPCSI), 20 August 1955 University El-Hadaeik Road, P.O. Box 26 Skikda 21000 Algeria; c Laboratory LGCES, Faculty of Technology, 20 August 1955 University Skikda, 2100 Algeria; d Department of Sciences and Technology, Faculty of Technology, 20 August 1955 University El-Hadaeik Road, P.O. Box 26 Skikda 21000 Algeria; e University 20 August 1955, Department of Nature and life Sciences Skikda, 21000 Algeria; f Laboratory of Ecobiology of Marine and Coastal Environments (EMMAL), Annaba University 23000 Algeria; g Laboratoire de Génie des Procédés pour le Développement Durable et les Produits de Santé (LGPDDPS), Département Génie des Procédés, Ecole Nationale Polytechnique de Constantine Algeria; h Izmir Katip Celebi University, Faculty of Fisheries, Department of Aquaculture Izmir Turkiye; i Scientific and Technical Research Center in Physical and Chemical Analysis (CRAPC) BP 384 Bou-Ismail RP 42004 Tipaza Algeria; j Research Unit in Physico-Chemical Analysis of Fluids and Soils (URAPC-FS) 11 Chemin Doudou Mokhtar, Ben Aknoun 16028 Alger Algeria; k Technical Platform for Physico-chemical Analysis (PTAPC-Bejaia) Targa Ouzemmour 06000 Bejaia Algeria; l Biotechnology Research Center (CRBt) Nouvelle Ville Ali Mendjli UV03 Constantine 25000 Algeria

## Abstract

*Cistus monspeliensis* L., a Mediterranean plant rich in polyphenolic compounds, was employed as a sustainable reducing and capping agent in the green synthesis of Ag/Ag_2_O/γ-Fe_2_O_3_ nanoparticles. The nanoparticles were characterized using UV-Vis spectroscopy, DRS, FTIR, XRD, FESEM-EDX, TEM, and zeta potential analyses. They were subsequently applied for the photocatalytic degradation of Crystal Violet (CV) under UVA and solar irradiation, as well as for evaluating their cytotoxicity against human colorectal cancer cells (HCT-116). The obtained NPs were polydisperse and nearly spherical with sizes averaging 26.49 ± 6.1 nm. Optical characterization using UV-Vis and DRS revealed an absorption peak at 274 nm and a direct bandgap value of 1.934 eV, enabling sufficient visible-light absorption. FE-SEM demonstrated a rough surface with visible particle aggregation, while EDX confirmed the elemental composition of the material. Ag/Ag_2_O/γ-Fe_2_O_3_ NPs exhibited high colloidal stability with a zeta potential of −46.9 mV. Owing to the synergistic combination between Ag and Fe, the nanomaterial achieved rapid photocatalytic degradation of CV (10 mg L^−1^ and a catalyst dosage of 0.5 g L^−1^), with removal efficiencies of 97.87% under UVA irradiation and 95.50% under solar irradiation within 60 minutes. Radical scavenger tests indicated that ˙OH and O_2_˙^−^ were the main reactive species involved in CV degradation. In addition, Ag/Ag_2_O/γ-Fe_2_O_3_ NPs presented potent cytotoxicity against HCT-116 cells; the activity was dose-dependent with an IC_50_ value of 23.34 ± 1.61 µg mL^−1^ using the MTT assay, inducing severe alterations indicative of apoptotic cell death and cytoskeletal disruption. These findings highlight the interdisciplinary potential of biosynthesized Ag/Ag_2_O/γ-Fe_2_O_3_ NPs in environmental remediation and biomedical applications.

## Abstract 2

Green-synthesized Ag/Ag_2_O/γ-Fe_2_O_3_ nanoparticles using *Cistus monspeliensis* leaf extract exhibit remarkable multifunctionality, achieving rapid photocatalytic degradation of crystal violet and potent cytotoxicity against HCT-116 cancer cells.

## Graphical abstract

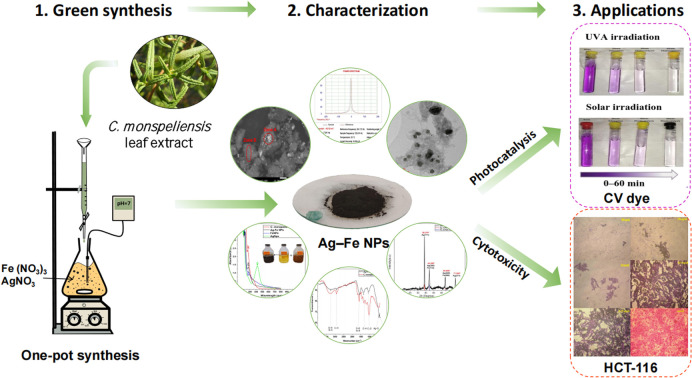

## Introduction

*Cistus monspeliensis* L., also known as Montpellier rockrose, is an evergreen shrub of the *Cistaceae* family that has, over time, been used traditionally in folklore medicine across the Mediterranean region, especially in Algeria, Tunisia, and Morocco, a region characterized by considerable botanical diversity.^1–3^*C. monspeliensis* leaves and their infusions were used for the treatment of arthrosis,^4^ pain relief,^5^ wound healing,^5^ asthma,^3^ infections,^1^ and diabetes.^6^ The medicinal value and high usage of this plant have been associated with its rich content of phytochemicals. Evaluation of *C. monspeliensis* phytochemical extracts of both the aerial parts and roots indicates a high occurrence of phytochemical families like polyphenols, flavonoids, ellagitannins, and tannins.^5^ Plants belonging to the Cistus genus, including *C. monspeliensis*, have been reported to have antioxidant,^7^ anti-inflammatory,^4^ analgesic,^4^ anti-genotoxic properties,^5^ and corrosion inhibition potential,^8^ depending on the extraction method and solvents used, in addition to significant antimicrobial and cytotoxic activities.^1,9^

As an evergreen shrub, *C. monspeliensis* provides year-round biomass availability, making it a practical and scalable resource; moreover, its leaves are rich in redox-active phytochemicals that can potentially support reduction and stabilization processes during nanoparticle formation.

Historically, plants and their extracts were valued for their therapeutic properties. More recently, they have gained attention in nanotechnology and have been explored in the green synthesis of metal-based nanoparticles (NPs).^10^ In this context, plant-derived phytochemicals play a dual role, where they can be used as natural reducing agents, by reducing metal ions into elemental NPs through redox reactions, and as stabilizers that prevent aggregation of the resulting NPs.^11,12^ Such capping/stabilization can influence nanoparticle size, surface chemistry, and colloidal stability, which are key factors governing biological and catalytic performance. This method of synthesis avoids the use of toxic chemicals and high energy consumption, often employed by conventional physico-chemical methods of nanoparticle synthesis, making the green synthesis route more affordable and eco-friendly, as it involves natural resources, such as plant extracts and micro-organisms.^12–14^

The synthesis of NPs is of great significance because of their inherent properties, considering they have at least one dimension ranging from 1 nm to 100 nm. Due to their high surface area and reactivity, NPs have distinctive physical, chemical, optical, electrical, and biological properties,^12,15^ and possess some remarkable properties that are not present or may be enhanced compared to their bulk counterparts.^16^ Their unique properties allow them to be engineered for specialized functions in numerous applications, including cancer treatment, targeted drug delivery, and imaging (Theranostics), adsorption, catalysis, sensing, and energy.^17,18^

Among green-synthesized metallic NPs, silver nanoparticles (AgNPs) have been widely explored for their potent antimicrobial activity against drug-resistant micro-organisms and their proven anticancer properties.^19–21^ Iron oxides (IONPs) in their different structures are renowned for their stability and catalytic activity, especially phases like hematite (α-Fe_2_O_3_), and some for their magnetic capabilities such as maghemite (γ-Fe_2_O_3_) and magnetite (Fe_3_O_4_). IONPs are economical, eco-friendly, abundant, and are generally regarded as relatively low-toxicity materials;^13,22^ they possess semiconductor properties and have an appropriate band gap energy ranging from 2.0–2.3 eV, which is suitable for efficient light absorption needed for applications like solar photocatalysis.^23^

The design and synthesis of NPs for multifunctional applications is often achieved through creating nanocomposites or bimetallic nanoparticles (BMNPs), which can lead to enhanced performance, greater efficiency, and improved economic viability.^24,25^ Ag/Fe-based NPs have been proposed as a means of combining the established antimicrobial properties of silver with iron's catalytic and magnetic properties. The goal is to reduce the cost of AgNPs while retaining, or even improving, silver's performance due to synergistic effects with catalytic and cheaper iron oxides.^13^ NPs containing both silver and iron/iron oxides have been synthesized using bio-methods based on different plant parts and micro-organisms. For example, ginger essential oil was used to fabricate Ag/Fe_2_O_3_ NPs with high antibacterial activity,^26^ while *Saussurea obvallata* leaf extract yielded Ag@Fe_2_O_3_ nanostructures with an echinus-like morphology (∼35 nm).^27^ Similarly, *Salvadora persica* bark extract produced core–shell α-Fe_2_O_3_@Ag and Fe_3_O_4_@Ag NPs (50–60 nm),^28^ and *Syzygium aromaticum* bud extract generated core–shell Ag–Fe BMNPs with near-spherical particles (∼16 nm).^11^ Other Ag/IONP hybrid nanostructures have also been reported using *Kulekhara* leaves,^29^*Carica papaya* peel extract,^13^*Passiflora edulis* leaf extract,^12^ beetroot extract,^30^ and fungal filtrates.^31^ Overall, many studies have shown that hybrid systems can outperform monometallic counterparts in both biomedical and environmental applications.^24,25^ Despite the large number of green-synthesized Ag/iron-oxide hybrids reported, many studies remain application-specific, and the combined evaluation of environmental photocatalysis together with biological screening within the same green-synthesized material is still limited.

Pure biosynthesized AgNPs and IONPs have already been studied and successfully utilized for inhibiting cell growth of HCT-116 colorectal cancer, with IC_50_ values as low as 5 µg L^−1^ for AgNPs, indicating a strong cytotoxic effect.^20,32^ HCT-116 cells were selected as a relevant colorectal cancer model given the increasing burden of colorectal cancer, particularly the rise in early-onset cases (<50 years), with incidence nearly doubling in the U.S. since the early 1990s and increasing across multiple countries, as summarized by Dharwadkar *et al.*^33^ However, plant-mediated Ag–iron oxide hybrid nanostructures remain comparatively underexplored in cytotoxicity studies, especially against HCT-116 cells. Importantly, combining Ag with iron oxides can yield a multifunctional platform, where Ag may enhance biological activity while the iron-oxide component provides a stable semiconductor/catalytic matrix; therefore, evaluating both biomedical and environmental performance in one eco-friendly material is well motivated. Ag/Fe-based NPs have also been widely applied for the photocatalytic degradation of dyes, often achieving near-complete removal.^13,34,35^ Crystal violet was selected as a model dye pollutant because it is widely used in several industries, is chemically stable and persistent, and is associated with toxic effects; therefore, it provides a stringent target for evaluating photocatalytic performance.^36^

In this work, we followed a cost-performance design strategy, where iron oxides provide an abundant, low-toxicity, and catalytically active platform, while a small silver fraction is introduced to enhance functionality. Accordingly, we investigate whether a plant-mediated Ag/iron-oxide hybrid can achieve efficient crystal violet removal under light irradiation while maintaining measurable cytotoxic activity against HCT-116 cells. Herein, we report the green synthesis of Ag/Ag_2_O/γ-Fe_2_O_3_ nanoparticles using *Cistus monspeliensis* leaf extract as a bioreductant and stabilizing agent, and we evaluate their dual functionality for (i) water-treatment remediation *via* photocatalytic degradation of crystal violet and (ii) proof-of-concept anticancer screening against HCT-116 cells. To the best of our knowledge, *C. monspeliensis* has not previously been reported for nanoparticle synthesis, and this is the first study to assess an Ag/Fe-based hybrid system for crystal violet photocatalysis together with HCT-116 cytotoxicity within a single green-synthesized platform.

## Materials and methods

### Materials

The metal precursors used were iron (iii) nitrate nonahydrate (Fe(NO_3_)_3_·9H_2_O, ≥98%) and silver nitrate (AgNO_3_, ≥99%), both are of ACS reagent grade and obtained from Sigma-Aldrich. Sodium hydroxide (NaOH, ≥98%) and ethanol (96%) both of analytical grade, were purchased from Prochima Sigma. Hydrochloric acid (HCl, reagent grade, 36.5–38%, Honeywell). Crystal Violet (AR grade) from TMMEDIA. Distilled water (DW) was used as solvent throughout the study. *Cistus monspeliensis* fresh leaves were collected from Ramdane Djamel province, Skikda city (North-eastern Algeria).

### Plant preparation and leaf extraction

*C. monspeliensis* fresh leaves were collected during vegetative (pre-flowering) stage, to minimize variability in phytochemical composition, the leaves were washed thoroughly with distilled water to remove impurities, they were then shade-dried and ground into fine powder to increase the surface area. 30 g of leaf powder was suspended in 0.5 L of DW, heated for 30 min at 80 to 90 °C, and then filtered through Whatman paper No. 4. The pH of the extract was measured to be 5.1, it was then stored in the dark at 4 °C.^37^

### Synthesis of Ag/Ag_2_O/γ-Fe_2_O_3_ NPs

**Fig. 1:**
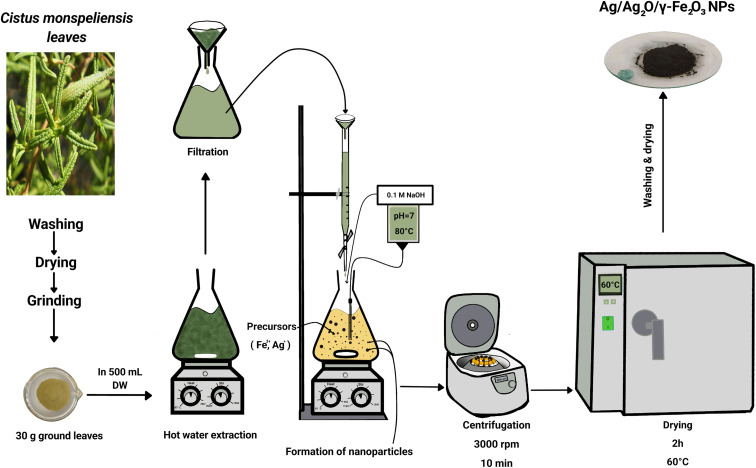
Graphic representation of key steps in the green synthesis of Ag/Ag_2_O/γ-Fe_2_O_3_ NPs using *C. monspeliensis* leaf extract.

The biosynthesis method was done following a modified protocol by Al-Zahrani *et al.*^38^ Using a burette, 0.1 L of *C. monspeliensis* aqueous leaf extract was added drop by drop to an Erlenmeyer flask containing a mixture containing 1 g of Fe(NO_3_)_3_·9H_2_O and 0.1 g of AgNO_3_ in 0.1 L DW, the temperature was maintained between 70 and 80 °C, stirring at 1000 rpm for two hours, and pH was adjusted to 7 using 0.1 M NaOH (The pH was maintained at 7 using dropwise addition of 0.1 M HCl when necessary). Under these aqueous, near-neutral and air-exposed green-synthesis conditions, Fe-based species are expected to evolve toward iron oxide-type phases with time,^39^ while AgNO_3_ can yield metallic Ag and/or silver oxide in plant-mediated synthesis,^40^ consistent with the phases identified by XRD. The full procedure is shown in Fig. 1.

Because measurements were carried out through shared facilities with waiting queues, the time between synthesis and characterization could extend to several days. Therefore, unless otherwise stated, all analyses and application experiments reported were performed on the stored nanoparticle batch.

### Characterization of the NPs

To characterize the properties of the biosynthesized Ag/Ag_2_O/γ-Fe_2_O_3_ NPs using *C. monspeliensis*, a range of analytical methods was employed. Optical properties in colloidal suspension were studied using UV-Vis spectroscopy (Shimadzu UV-1900-i, Japan), by observing the absorption peaks between 200 and 800 nm, using 10-fold dilution, before scanning using quartz cuvettes, while the bandgap energy was estimated through diffuse reflectance spectroscopy for solid samples (UV-DRS, Thermo Scientific Evolution 201, USA). The structure, crystallinity and average crystallite size were assessed by Powder X-ray diffraction (PXRD, Empyrean 3rd gen, Malvern Panalytical, Netherlands), using Cu Kα (*λ* = 0.15406 nm), scanning 2*θ* angles between 20° and 80°. The measurements were performed in gonio scan mode using a diffracted-beam monochromator to suppress fluorescence/background and improve peak definition. The functional groups derived from the plant's phytochemicals were detected by conducting Fourier-transform infrared spectroscopy (FTIR, Shimadzu IRSpirit-X, Japan) coupled with an ATR accessory, in the range of 400–4000 cm^−1^. Morphology and elemental components were assessed by Field emission-scanning electron microscopy (FE-SEM, JEOL JSM-7200F, Japan), equipped with a Backscattered electron detector for compositional contrast (BED-C) and energy-dispersive X-ray spectroscopy (EDX), the Ag/Ag_2_O/γ-Fe_2_O_3_ NPs were put onto a carbon tape with an aluminum holder. The particle size, shape, and dispersion were provided by Transmission Electron Microscopy (TEM) micrographs at various magnifications (JEOL JEM-F200, Japan), by preparing a colloidal ethanol suspensions of 0.01–0.1 mg mL^−1^ of Ag/Ag_2_O/γ-Fe_2_O_3_ NPs, followed by sonication for 5 minutes, one drop was deposited on a copper/carbon 200 mesh grid. Finally, zeta potential (ZP) measurements using a NICOMP 380 ZLS analyzer (USA), with a HeNe laser, a suspension of 40 mg mL^−1^ was prepared in ultra-pure water (Millipore, Merck, Germany) and then sonicated for 5 minutes, which revealed their colloidal stability and surface charge.

### Photocatalytic degradation study

To evaluate the photocatalytic degradation of the bioproduced Ag/Ag_2_O/γ-Fe_2_O_3_ NPs under different irradiation sources: solar (sunlight experiments were conducted around solar noon (≈12:00 local time) in Skikda, Algeria. Solar position calculations (SOLPOS) indicate a peak extraterrestrial global horizontal irradiance of ∼1.07 kW m^−2^ at this time, confirming operation under maximum solar incidence during clear weather days) and UVA light using a lamp (*λ*_max_ = 395 nm, ∼3.138 eV) positioned approximately 10 cm above the thermostable beaker, Crystal Violet (CV) dye solutions of 10, 20, 30, 50 and 70 mg L^−1^ were prepared using distilled water. The experiments were conducted for 90 minutes (30 minutes of adsorption in the dark followed by 60 minutes of light irradiation), using 0.5 g L^−1^ of Ag/Ag_2_O/γ-Fe_2_O_3_ NPs, with constant initial conditions: pH (natural), temperature (20 ± 1 °C), and stirring speed (300 rpm). Samples taken at different time stamps (0, 5, 10, 20, 30, 40, 50, 60 minutes) were centrifuged for 10 minutes, and their absorbance was measured against a blank without the dye at *λ*_max_ = 578 nm. Degradation spectra for both solar and UVA irradiation were scanned from 400–700 nm at 30 mg L^−1^. Photolysis (without the NPs) and adsorption (in the dark) experiments were assessed to compare their individual influence on photocatalysis.

The degradation rate was calculated using the eqn (1):1
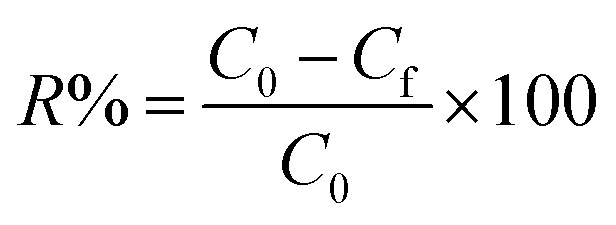
where: *C*_0_ is the initial concentration before light irradiation, and *C*_f_ is the final concentration at 60 min.

Eqn (2) was used to study the kinetics using the non-linear equation of pseudo-first order (PFO) model, as it allows for a more accurate estimation of the apparent rate constant:^41,42^2
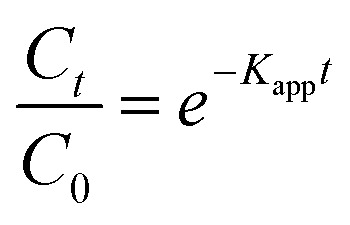
where: *C*_*t*_ is the concentration (mg L^−1^) at any given time *t*, C_0_ is the initial concentration (mg L^−1^), *K*_app_ is the apparent rate constant of the PFO kinetics.

### Anti-cancer activity by MTT assay

The cytotoxicity of the green-synthesized Ag/Ag_2_O/γ-Fe_2_O_3_ NPs was determined through the MTT [3-(4,5-dimethylthiazol-2-yl)-2,5-diphenyltetrazolium bromide] assay using standard protocols.^43^ HCT-116 human colon carcinoma cells from the American Type Culture Collection (ATCC, Rockville, MD, USA), were cultured in RPMI-1640 medium, supplemented with 10% heat-inactivated fetal bovine serum and 50 µg mL^−1^ gentamycin, the cells were incubated at 37 °C in a 5% CO_2_ humidified atmosphere and were regularly subcultured. For the assay, the cells were seeded with a density of 5 × 10^4^ cells per well in 96-well tissue culture plates and grown for 24 hours before treatment with serial dilutions of Ag/Ag_2_O/γ-Fe_2_O_3_. Cells were further incubated for a period of 24 hours after exposure to different concentrations of Ag/Ag_2_O/γ-Fe_2_O_3_ NPs (500, 250, 125, 62.5, 31.25, 15.6, 7.8, 3.9, 2, 1, and 0 µg mL^−1^) and incubated for an additional 24 hours. After treatment, MTT reagent was added, and cells were incubated, insoluble formazan crystals formed by the reduction of the MTT reagent were then dissolved by using 50 µL DMSO and the optical density (OD) was measured at 590 nm using a SunRise microplate reader (TECAN Inc.,USA). The % viability of the cells was calculated using eqn (3):3
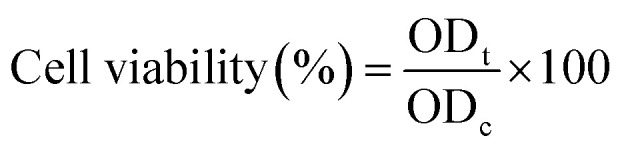
where OD_t_ is the mean optical density of the treated wells and OD_c_ is that of the untreated control. The IC_50_ value, representing the concentration of Ag/Ag_2_O/γ-Fe_2_O_3_ NPs needed to decrease the viability of the cells by 50%, was calculated from the dose–response curve using GraphPad Prism software (San Diego, CA, USA).

## Results and discussion

### UV-vis spectroscopy

**Fig. 2:**
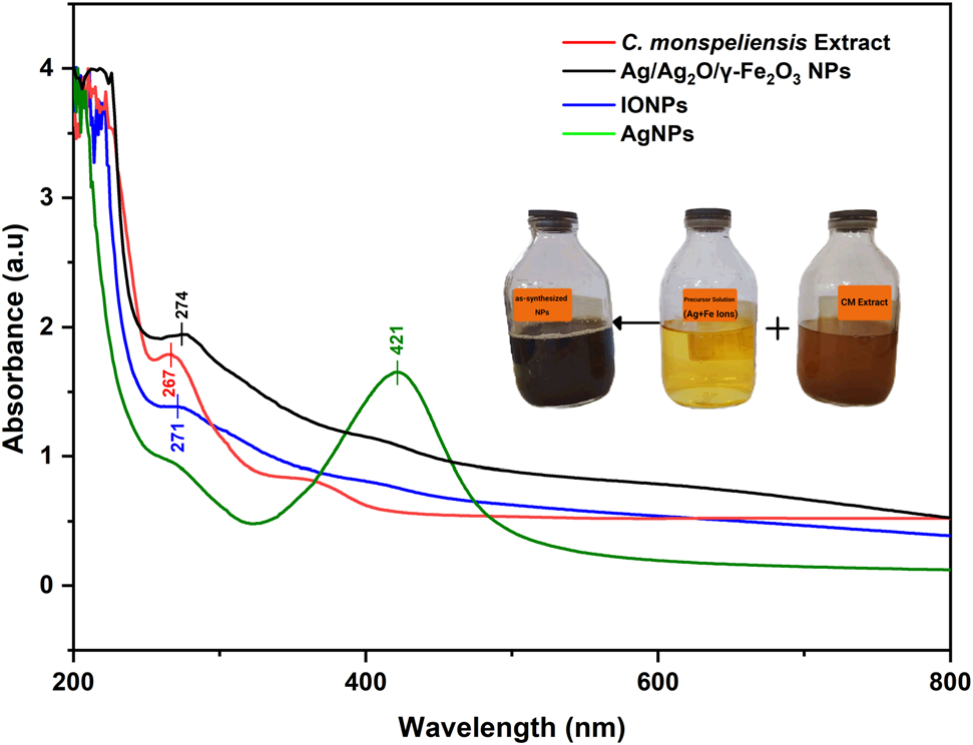
UV-Vis absorption spectra of *C. monspeliensis* aqueous extract, and the as-synthesized Ag/Ag_2_O/γ-Fe_2_O_3_ NPs, IONPs, and AgNPs.

The successful synthesis of the Ag/Ag_2_O/γ-Fe_2_O_3_ NPs was first confirmed visually by the color change of the precursor solution. Upon the addition of the aqueous plant extract, the color switched from golden-yellow to brownish–black which indicates the formation of Ag/Ag_2_O/γ-Fe_2_O_3_ NPs. UV-Vis spectroscopy was then employed for verifying nanoparticle formation through the detection of characteristic absorbance peaks. The UV-Vis absorption spectra shown in Fig. 2 of the *C. monspeliensis*, and the as-synthesized Ag/Ag_2_O/γ-Fe_2_O_3_ NPs, iron oxide nanoparticles (IONPs), and AgNPs showed distinct peaks of the different compositions. The plant extract itself had an absorption peak at 267 nm, which is characteristic of phenolics or flavonoids,^44^ which is consistent with the well-reported prevalence of aromatic phytochemicals in *Cistus monspeliensis*.^45^ Moreover, a broad shoulder between 300 and 380 nm is consistent with the absorption range for conjugated flavonoid and other polyphenolic compounds.^44^

IONPs displayed a weak shoulder at 271 nm, indicating absorbance attributed to iron species or Fe–phytochemical capping agent interactions. This result is within the characteristic absorption range of iron oxide nanoparticles (IONPs) between 250 and 350 nm.^46^ AgNPs, on the other hand, displayed a surface plasmon resonance (SPR) peak at 421 nm, indicating the synthesis of AgNPs. It falls within the widely reported SPR range for biosynthesized AgNPs (380–580 nm),^24^ which is dependent on particle size, shape, and surface chemistry.^46^

Notably, the Ag/Ag_2_O/γ-Fe_2_O_3_ NPs displayed a clear peak at 274 nm, also within the absorption range of iron-based NPs, and agree with literature reports of green-synthesized Fe_*x*_Oγ NPs that showed peaks at 270 nm,^47^ 277 nm,^48^ and 294 nm,^49^ but no characteristic Ag SPR band close to 420 nm. This lack might be due to Fe surface coverage, plasmon damping effects, or a possible core–shell structure with Ag encapsulated by γ-Fe_2_O_3_.^11,24^ Such inhibition of the Ag SPR signal was reported in earlier studies in hybrid green synthesized systems containing both Ag and Fe.^24,50^ Herein, the high concentration of Fe ions compared to Ag ions in this study (10 : 1 mass ratio) has possibly led to Fe-dominant surfaces, causing optical masking of silver. Despite the absence of a SPR peak, it is important to note that the Ag/Ag_2_O/γ-Fe_2_O_3_ nanoparticles exhibit significant absorption in the UV-Vis region, higher than the individual IONPs alone due to the addition of silver.

### Diffuse reflectance spectroscopy (DRS)

Diffuse Reflectance Spectroscopy (DRS) is an important technique used to investigate the optical properties of materials, it was applied to the green synthesized Ag/Ag_2_O/γ-Fe_2_O_3_ NPs in powdered form to investigate their visible light absorption by measuring the bandgap energy, which is a fundamental parameter to assess whether Ag/Ag_2_O/γ-Fe_2_O_3_ NPs can absorb light in the visible region, and therefore could be applied as photocatalysts under sunlight.^51^

**Fig. 3:**
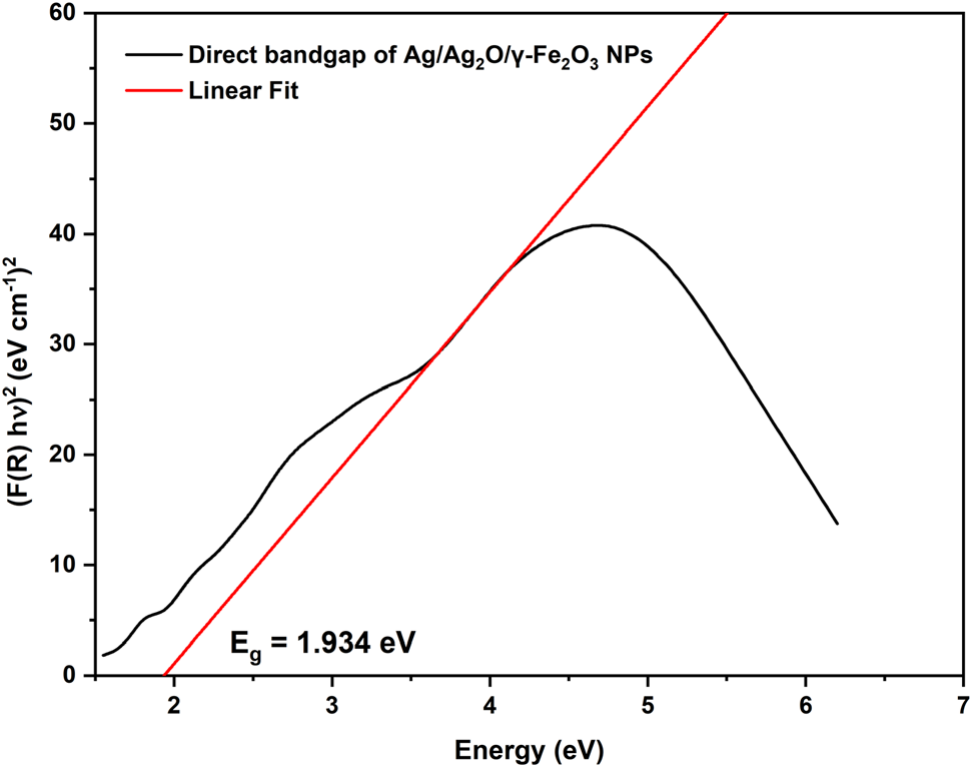
Determination of the direct bandgap energy of Ag/Ag_2_O/γ-Fe_2_O_3_ NPs from the Tauc plot.

The Kubelka–Munk function (eqn (4)) was used to convert the diffuse reflectance (*R*) data obtained from a UV-Vis spectrophotometer, into a quantity proportional to the absorption coefficient, which is important to estimate the direct bandgap energy from the Tauc plot.^14^4
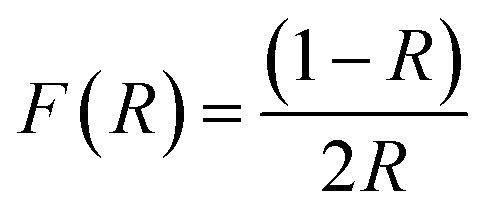
where *R* is the reflectance, Fig. 3 represents the Tauc plot ([*F*(*R*)*hν*]^2^*vs. hν*), where a linear fit was applied to its most linear region, the extrapolation of this line to the *X*-axis revealed the direct bandgap energy of Ag/Ag_2_O/γ-Fe_2_O_3_ NPs to be 1.934 eV.

Previous studies reported similar results, as A *et al.* reported a reduction in the bandgap energy upon the addition of silver to Fe_2_O_3_ NPs. For pure Fe_2_O_3_ NPs, the bandgap energy was 2.01 eV, and it shifted to 1.93 eV in the Fe_2_O_3_/Ag nanocomposites, which matches our findings for the Ag/Ag_2_O/γ-Fe_2_O_3_ NPs.^14^ Endres *et al.* also found that the addition of silver to the structure, results in a reduction of the direct bandgap energy, with a shift from 3.34 eV of the pure Fe_2_O_3,_ to a lower value of 2.91 eV for Ag/Fe_2_O_3_ nanocomposites.^25^

The presence of AgNPs in Ag/Fe-based nanocomposites can enhance the absorption of visible light by the NPs, due to the surface plasmon resonance (SPR) of silver and narrowing of the bandgap energy, it can also inhibit the electron–hole recombination and improve charge carrier separation efficiency.^51^ Therefore, our measured direct bandgap energy of *E*_g_ = 1.934 eV showcases the potential of the Ag/Ag_2_O/γ-Fe_2_O_3_ NPs as good photocatalysts under the sunlight.

### Fourier-transform infrared spectroscopy (FT-IR)

**Fig. 4:**
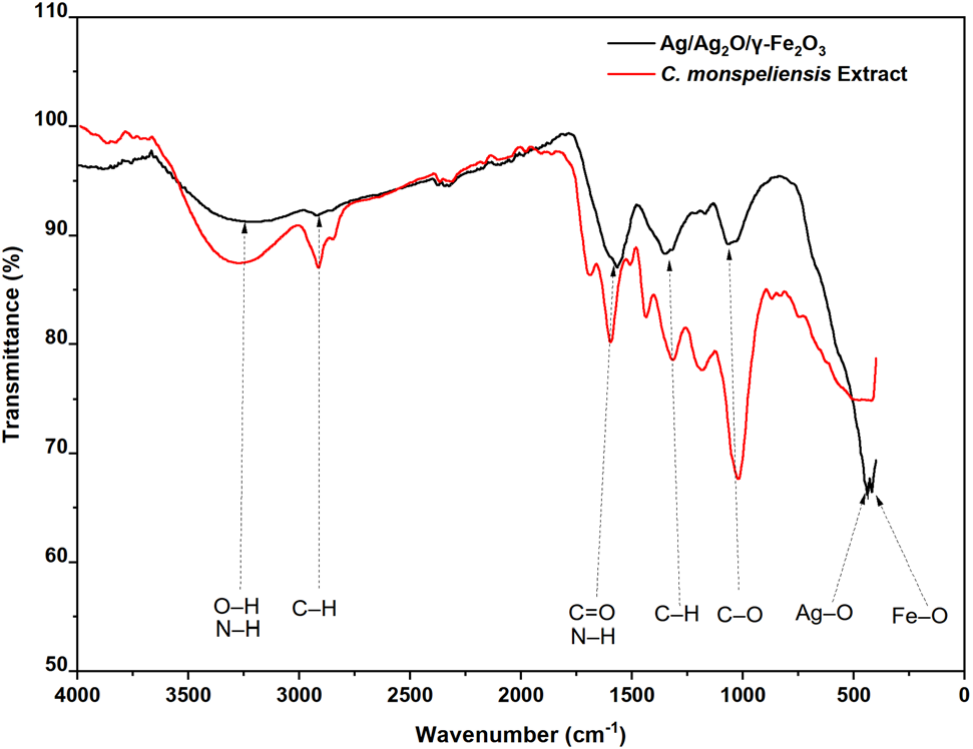
FTIR Spectra of the aqueous extract of *C. monspeliensis* and Ag/Ag_2_O/γ-Fe_2_O_3_ NPs.

The FTIR spectra of both the dried aqueous extract of *C. monspeliensis* and Ag/Ag_2_O/γ-Fe_2_O_3_ NPs shown in Fig. 4, exhibited distinct peaks relating to the functional groups found in the plant extract and bound to the NPs. A broad peak in the range between 3000 and 3500 cm^−1^ corresponds to O–H stretching vibrations of alcohols or N–H stretching of phenolics,^52^ peaks for aliphatic C–H stretching of methylene groups at 2923 and 2860 cm^−1^,^53^ carbonyl stretching C=O at 1699 cm^−1^, N–H bending vibrations of amines at 1607 cm^−1^,^26^ aliphatic C–H bending at 1441 cm^−1^,^54^ and a peak at 1321 cm^−1^ and 1030 cm^−1^ for C–O stretching of alcohols or ethers.^55^ These peaks indicate the presence of polyphenols, flavonoids, carbohydrates, and proteins.^10,25,53^ For the Ag/Ag_2_O/γ-Fe_2_O_3_ NPs, some peaks revealed shifts reflecting hydrogen bonding and interaction with the surface of the NPs,^26^ the C=O/N–H region shifted from 1699 and 1607 to 1567 cm^−1^, reflecting involvement in reduction and capping processes,^10^ moreover, the peaks at 1441 and 1321 cm^−1^ shifted to a single peak at 1356 cm^−1^, and the band at 1030 cm^−1^ shifted to 1070 cm^−1^, reflecting changes in C–H and C–O related functional groups. Significantly, new peaks at 440 and 418 cm^−1^ corresponding to Ag–O and Fe–O bonds, respectively, confirm the successful synthesis of Ag/Ag_2_O/γ-Fe_2_O_3_ NPs; similar peaks of 445 and 420 cm^−1^ for Ag–O and Fe–O bonds, respectively, were found in the study of green synthesized Ag@Fe_2_O_3_ NPs by Jadhav *et al.*^34^ Overall, the band shifts and the appearance of metal–oxygen vibrations provide strong evidence that plant biomolecules participate in metal-ion reduction and stabilize the resulting nanoparticles.^46^

### X-ray diffraction (XRD)

**Fig. 5:**
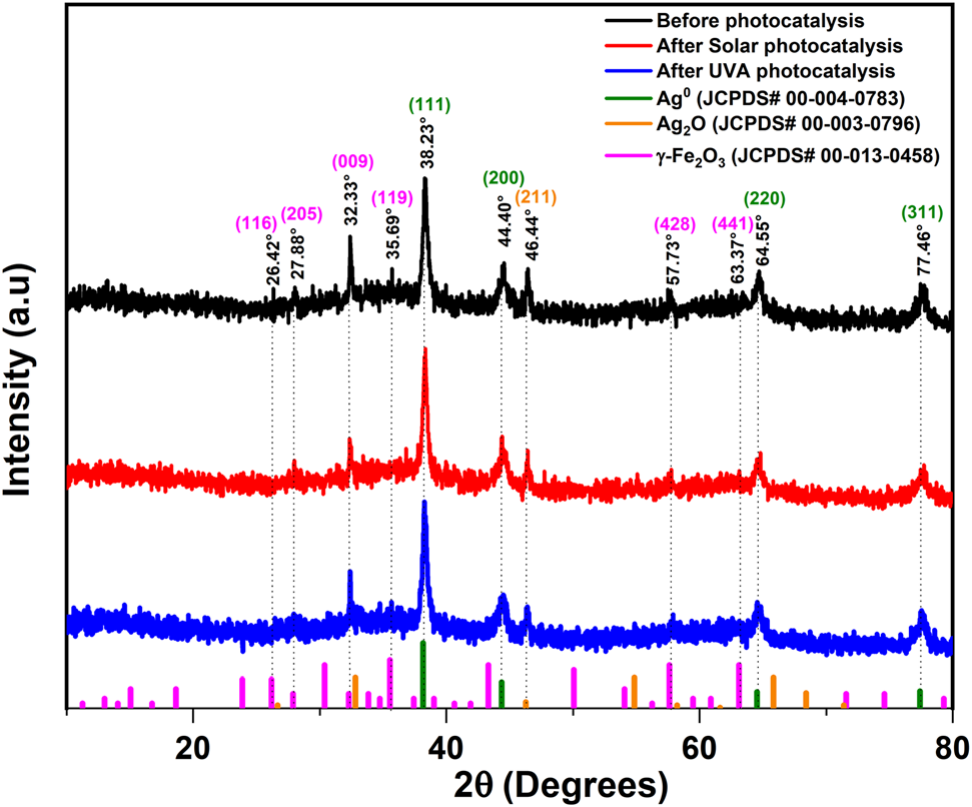
XRD Pattern of the green synthesized Ag/Ag_2_O/γ-Fe_2_O_3_ NPs before and after photocatalysis.

X-ray diffraction (XRD) is a key technique for determining the crystalline phases, structure, and structural stability of the synthesized nanoparticles (NPs), both before and after photocatalysis. Fig. 5 presents the XRD pattern of the green-synthesized Ag/Ag_2_O/γ-Fe_2_O_3_ NPs prepared using *C. monspeliensis* extract, together with the corresponding reference JCPDS cards.

The diffractogram shown in Fig. 5 (before photocatalysis) corresponds to the stored batch used in the photocatalytic experiments, since the nanoparticles were not applied immediately after synthesis and drying. Under non-inert storage conditions, including possible exposure to air and moisture during drying, storage, and repeated handling, gradual oxidation and structural reorganization is expected, particularly for Fe-containing systems. Fe^0^, if initially present, is unstable and can progressively transform into iron oxide phases over time.^39,56^ Consistent with this, the powder XRD recorded shortly after synthesis showed the reflections commonly reported for freshly prepared Ag–Fe systems, with dominant Ag^0^ peaks and potential overlapping Fe-related contributions. In contrast, the stored sample exhibits clear γ-Fe_2_O_3_ (maghemite) reflections alongside Ag^0^ and a weak Ag_2_O contribution. For transparency, a direct comparison between the initial pattern and the same batch after storage is provided in the SI (Fig. S1).

The diffractogram in Fig. 5 (before photocatalysis) shows the characteristic reflections of metallic silver (Ag^0^) at 2*θ* = 38.23°, 44.40°, 64.55°, and 77.46°, assigned to the (111), (200), (220), and (311) planes of Face-centered cubic structure (FCC), in agreement with the standard silver pattern (JCPDS No. 00-004-0783).^57^ A weak reflection near 46.44° (211) is consistent with cubic Ag_2_O (JCPDS No. 00-003-0796),^58^ while the majority of the remaining peaks, including ones appearing at 26.42°(116), 27.88°(205), 32.33°(009), 35.69°(119), 57.73°(428), and 63.37°(441), are attributed to γ-Fe_2_O_3_ (maghemite) (JCPDS No. 00-013-0458).^59^

After photocatalysis under both solar and UVA irradiation, the prominent diffraction peaks remain at essentially the same 2*θ* positions with only minor shifts, indicating that the dominant crystalline phases were retained and no new crystalline phases observed within XRD detection limits.^60^ However, a slight decrease in intensity is observed for the γ-Fe_2_O_3_-related peaks at 26.42°, 32.33°, and 35.69°, which is commonly reported after photocatalytic runs and is usually linked to surface-related changes like microstrain instead of a complete phase change.^61^ In the literature, reduced peak intensities without major peak shifts are often explained by partial surface coverage from adsorbed dye molecules and degradation intermediates, which can mask diffracting domains and block active sites, and by changes in powder texture/preferred orientation introduced during catalyst recovery, washing, and re-drying, which can alter relative peak intensities even when the phase composition is unchanged.^61–63^ In addition, Ag_2_O-containing systems can undergo partial light-induced reduction from Ag_2_O to Ag^0^ during irradiation, which may slightly modify the intensity contribution in the Ag(111) region without necessarily generating new peaks or shifting the Ag reflections.^64^

The crystallite size of the nanoparticles before and after photocatalysis was determined using the Scherrer equation (eqn (5)).^65^5
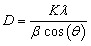
In the equation above, *D* is the average crystallite size, *K* is the Scherrer constant (0.9), is the X-ray wavelength (0.15406 nm for Cu-kα radiation), is the full width at half maximum (FWHM) of the diffraction peak in radians, and is the Bragg diffraction angle in radians.^65^ The estimated average crystallite size for all the samples remained essentially constant, with an average of 17.96 ± 6.55 nm before photocatalysis, 17.79 ± 6.53 nm after solar photocatalysis, and 17.51 ± 6.20 nm after UVA photocatalysis, supporting that the photocatalyst maintains its crystalline nature after the reaction and that the observed intensity changes are most consistent with surface adsorption/texture effects and minor surface modifications rather than a significant structural transformation.

### FESEM–EDX

**Fig. 6:**
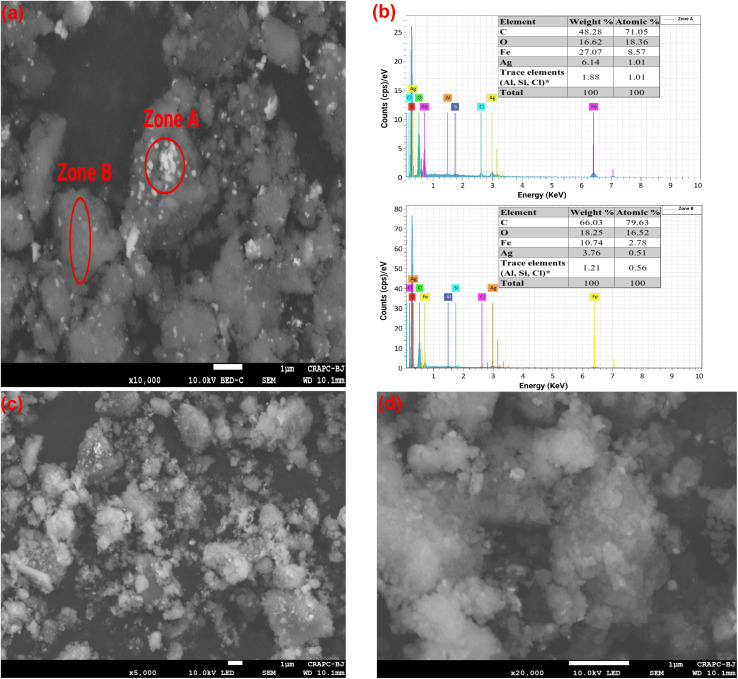
FE-SEM images at different magnifications of Ag/Ag_2_O/γ-Fe_2_O_3_ NPs: (a) ×10 000 magnification micrograph showing two distinct zones using BED-C detector, and (b) EDX spectra of both zones with elemental weight% and atomic%, (c and d) micrographs at ×5000 and ×20 000 magnifications using LED detector.

To study the morphology and surface structure of the biosynthesized Ag/Ag_2_O/γ-Fe_2_O_3_ NPs, we used Field Emission Scanning Electron Microscopy (FE-SEM) equipped with a Backscattered Electron Detector for compositional contrast (BED-C), which is sensitive to the average atomic number^66^ and a Low-Energy Detector (LED) for surface topographical contrast. Fig. 6a shows a heterogeneous structure with a rough surface with two contrasting regions, Zone A presents small, bright, near-spherical particles indicating heavy elements with a higher atomic number (*Z*), corresponding to both silver (47) and metallic iron (26), and the darker agglomerated particles in Zone B, possibly correspond to lighter elements from the plant material such as hydrogen (1), carbon (6) and oxygen (8).^67^Fig. 6c and d demonstrate a rough, sponge-like surface with an apparent porosity and significant agglomeration. Previous studies^51,68,69^ have reported a common tendency for agglomeration in green-remediated Ag–Fe NPs. TEM images, on the other hand, show well-dispersed NPs with no significant clustering, which indicates effective colloidal stabilization by the plant. Therefore, the agglomeration observed in FE-SEM is likely from the drying step, where the removal of the solvent causes the particles to cluster together due to hydrogen bonding, van der Waals interactions, and capillary forces to reduce total surface energy.^70,71^

EDX spectra in Fig. 6b of both Zone A (bright particles) and Zone B (dark particles with some bright spots) confirm individual peaks at 0.277 keV for carbon likely from the carbon tape and the biomolecules, and oxygen at 0.525 keV that can be attributed to the phytochemicals from *C. monspeliensis* extract and possibly due to metal oxidation in Ag_2_O and γ-Fe_2_O_3_ phases, iron (Fe) had multiple peaks at 0.7, 6.40 and 7.06 keV, and peaks for silver (Ag) at 2.98 and 3.15 keV, indicating successful synthesis of Ag/Ag_2_O/γ-Fe_2_O_3_ NPs. Silicon (Si) and chlorine (Cl) were also present in both zones in trace amounts, which could be attributed to insufficient washing of the plant or contamination during the synthesis (pH adjustment), and aluminum (Al) from the specimen stub of the instrument.

The presence of both metals in the Ag/Ag_2_O/γ-Fe_2_O_3_ NPs was confirmed by the FE-SEM/EDX analysis in the two regions (A and B), but in varying proportions. Zone A, which represents the brighter nanoparticle cluster, contained higher amounts for both metals, Fe (27.07 wt%, 6.14 at%) and Ag (8.57 wt%, 1.01 at%), while Zone B showed lower levels, Fe (10.74 wt%, 3.76 at%) and Ag (2.78 wt% and 0.51% at%), but was richer in C (66.03 wt%, 79.63 at%) and O (18.25 wt%, 16.52 at%).

### Transmission electron microscopy (TEM)

Transmission Electron Microscopy (TEM) is carried out on the synthesized Ag/Ag_2_O/γ-Fe_2_O_3_ NPs using *C. monspeliensis* aqueous extract as it allows the retrieval of key information such as: the visualization of the morphology, crystallinity, dispersion, and the accurate estimation of the size of the NPs.^72^

**Fig. 7:**
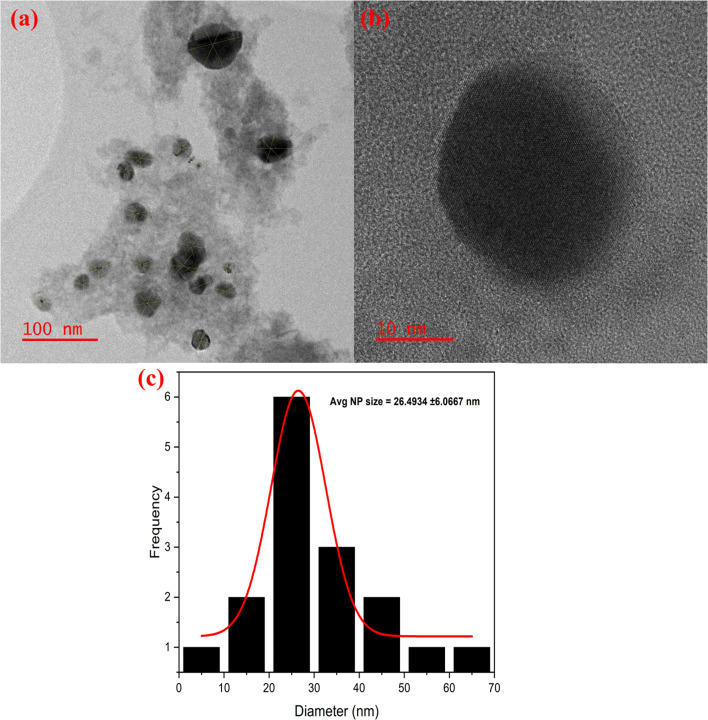
TEM images of Ag/Ag_2_O/γ-Fe_2_O_3_ NPs at different magnifications (a and b), and their size distribution (c).

The TEM micrograph Fig. 7a reveals a polydisperse distribution of mostly spherical particles with varying contrast lacking significant agglomeration, they are well-dispersed and embedded into an organic layer corresponding to the natural capping and stabilizing phytochemicals.^73^ These observed morphologies are consistent with plant-mediated NPs, where the complex nature of varying phytochemicals leads to uncontrolled nucleation and growth.^74,75^ The size of the NPs were measured using ImageJ software, each particle was measured three times, the size distribution analysis in Fig. 7c shows that the size of Ag/Ag_2_O/γ-Fe_2_O_3_ NPs ranges between 7 and 70 nm, with a dominant average diameter of 26.49 ± 6.1 nm. Similar results were found by Malik *et al.*, where they created Ag–Fe NPs using *Salvia officinalis* leaf extract; they reported polydispersed near-spherical NPs that have an average diameter of 27.48 ± 6.88 nm.^65^Fig. 7b represents one single isolated nanoparticle of 27.284 ± 1.66 nm, showing a dark core and a thin lighter shell. Although lattice-like fringes are visible upon zooming in, the image resolution was not sufficient to accurately determine the interplanar spacing (*d*-spacing) and thus phase assignment cannot be confirmed from TEM alone. Therefore, the core–shell architecture remains a possibility and would require High-Resolution TEM and complementary detectors/analyses such as EDX mapping, Annular Dark Field/Bright Field (ADF/BF, or Electron Energy Loss Spectroscopy (EELS) for confirmation. Overall, variations in size, shape, and contrast suggest that the Ag/Ag_2_O/γ-Fe_2_O_3_ system may be heterogeneous, potentially comprising core–shell-like particles, Ag–Fe nanocomposites, or separated Ag and Fe-based nanoparticles, which is commonly reported for phytomediated synthesis routes.^76^ For instance, core–shell structures have been reported by ref. 28 using *S. persica* and ref. 24 using *Gardenia jasminoides*. Dumbbell-shaped Fe–Ag NPs were obtained by ref. 52 from *Passiflora edulis*, while nanocomposite structures were reported by ref. 14 and 38, who synthesized Fe_2_O_3_/Ag nanocomposites using *Aloe vera* and *Buddleja lindleyana*, respectively.

The crystallite size calculated from XRD was smaller than the TEM estimated average; this difference may be due to the detection of XRD to only small crystals while missing larger aggregates, and the possibility that the NPs are polycrystalline or form clusters of multiple nanoscale crystallites.^65^

### Zeta potential

**Fig. 8:**
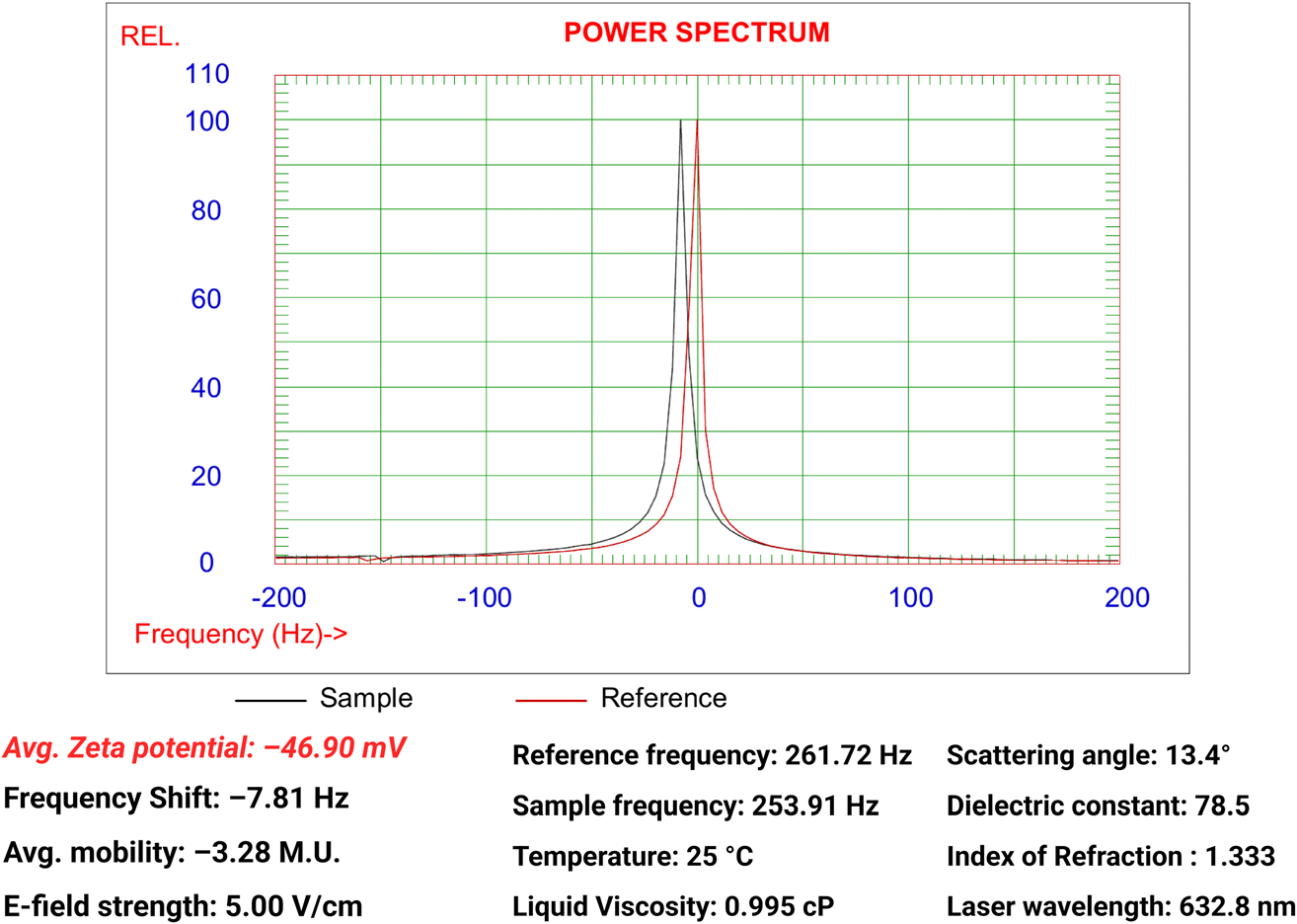
Zeta potential of Ag/Ag_2_O/γ-Fe_2_O_3_ NPs from the electrophoretic mobility peak provided by the power spectrum.

Zeta potential (ZP) serves as an indicator for the colloidal stability and dispersion of NPs; high absolute values above 30 mV reflect strong electrostatic repulsion between the particles, thus preventing aggregation and maintaining suspension stability.^77^ In our study, the zeta potential was calculated automatically by the instrument using the electrophoretic mobility peak in the power spectrum shown in Fig. 8 by applying the Smoluchowski model. The bio-fabricated Ag/Ag_2_O/γ-Fe_2_O_3_ NPs exhibited an average zeta potential of −46.90 mV with an average electrophoretic mobility of −0.000328 cm^2^ V^−1^ s^−1^, the highly negative surface charge is mainly derived from the adsorbed capping agents of *C. monspeliensis* on the surface of the NPs. This result is consistent with TEM observations, where good dispersion and lack of agglomeration was evident.

Previous studies reported a wide range of ZP measurements of biosynthesized Ag/Ag_2_O/γ-Fe_2_O_3_ NPs. Ag/Fe_2_O_3_ NPs made using beetroot extract showed a low potential of −0.2 mV,^10^ and using *Saraca asoca* leaf extract showed a moderate value of −10.42 mV,^51^ while Ag–Fe_3_O_4_ NPs using *Aegle marmelos* extract exhibited a higher potential of −41 mV.^78^ This difference in ZP values is due to the uncontrolled concentration of the stabilizing agents from varying phytochemicals across the different plants, also from other synthesis conditions: precursor type and concentration, pH, temperature, stirring speed, and duration of the reaction,^79^ which are not standardized in green synthesis methods using plant extracts. Our Ag/Ag_2_O/γ-Fe_2_O_3_ NPs are highly stable in dispersions and have a negative charge, which suggests their potential to be applied in fields like environmental remediation through electrostatic attraction with positively charged molecules like cationic dyes and heavy metal ions (*e.g.*, Pb^2+^, Hg^2+^, and Cd^2+^).

### Photocatalytic performance for the degradation of crystal violet

**Fig. 9:**
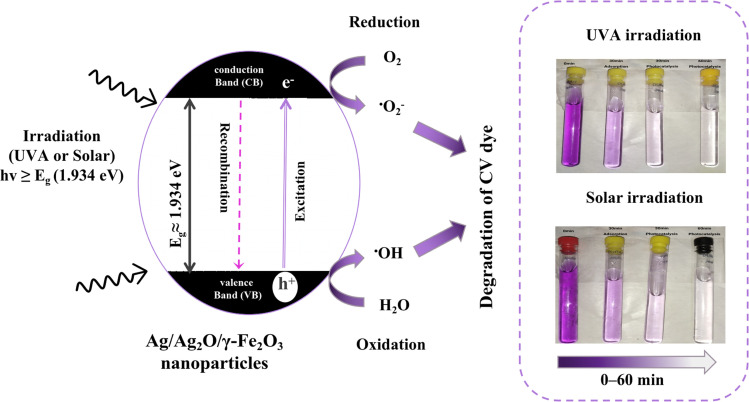
Illustration representing the mechanism of the photocatalytic degradation of CV dye using UVA and Solar irradiations. Experimental conditions: (*C* = 30 mg L^−1^, pH = natural of the solution, *V* = 200 mL, *m* = 0.1 g, *T* = 20 ± 1 °C).

Fig. 9 illustrates the fundamental mechanism of heterogeneous photocatalysis using the green-synthesized Ag/Ag_2_O/γ-Fe_2_O_3_ NPs (*E*_g_ = 1.934 eV) to degrade an organic dye (CV) using different irradiations (UVA and sunlight).

This advanced oxidation process starts by the irradiation of a semiconductor (Ag/Ag_2_O/γ-Fe_2_O_3_ NPs in this study) with photons that possess energy that meets or exceeds its estimated bandgap (*hν* ≥ *E*_g_).^80^ Upon the absorption of these photons, electrons in the valence band (VB) get excited and jump to the conduction band (CB), it generates electron–hole pairs (e^−^/h^+^) that start a series of reduction and oxidation reactions.^81^ Oxidation is driven by the holes (h^+^) at the VB, which react with water (H_2_O) or hydroxide ions (OH^−^) adsorbed on the surface of the material to produce hydroxyl radicals (˙OH). While the electrons at the CB react with dissolved oxygen (O_2_) to produce superoxide radical anions (O_2_˙^−^), these reactions generate reactive oxygen species (ROS) that exhibit strong oxidative potential, allowing the degradation of organic pollutants to harmless non-toxic by-products like water and CO_2_.^82^

To evaluate the photocatalytic performance, degradation kinetics were investigated under both solar and UVA irradiation (395 nm ∼3.138 eV). The energy from the sunlight and the UVA lamp should be sufficient for the generation of ROS, since the bandgap energy of the Ag/Ag_2_O/γ-Fe_2_O_3_ NPs was determined to be 1.934 eV *via* diffuse reflectance spectroscopy (DRS).

**Fig. 10:**
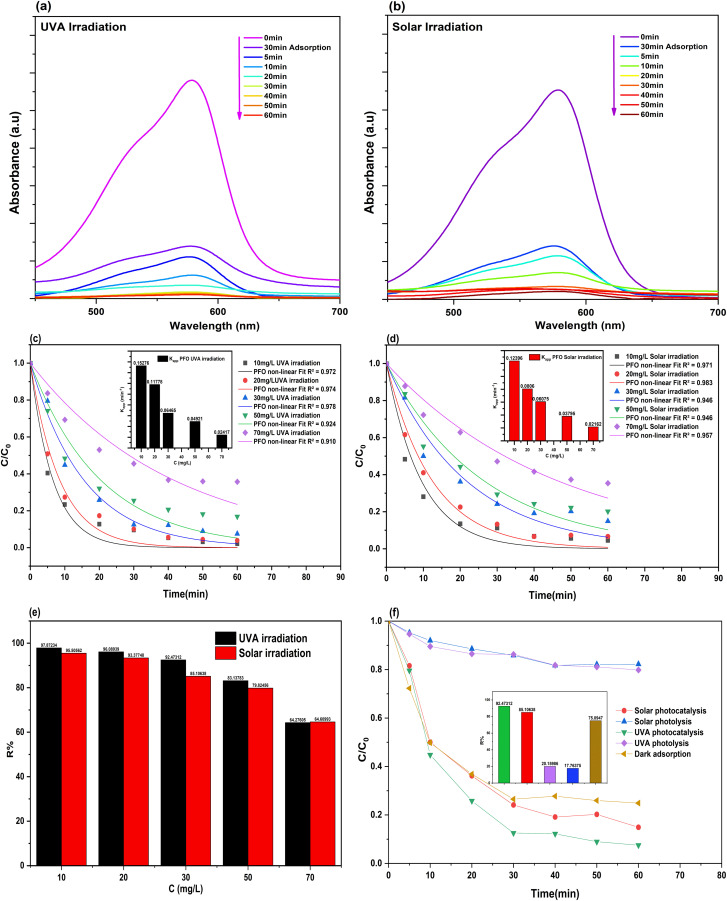
Photocatalytic degradation of crystal violet (CV) using Ag/Ag_2_O/γ-Fe_2_O_3_ NPs under UVA and Solar irradiation: (a and b) UV-Vis spectral evolution at 30 mg L^−1^; (c and d) effect of dye concentration (10–70 mg L^−1^) and their corresponding apparent rate constant (*K*_app_) based on PFO kinetics non-linear fitting (*R*^2^ > 0.9); (e) removal efficiency of CV at varying concentrations and light sources; (f) effect of irradiation (dark *vs.* light) and catalyst presence (photocatalysis *vs.* photolysis) at 30 mg L^−1^. Experimental conditions: (*C* = varying, pH = natural of the solution, *V* = 200 mL, *m* = 0.1 g, *T* = 20 ± 1 °C).

Fig. 10 represents a panel of figures (a–f) of the photocatalytic degradation of Crystal Violet dye. Fig. 10(a and b) exhibits the UV-Vis spectral evolution (90 min total), the decline in absorbance at the maximum wavelength (*λ*_max_ = 578 nm) during the initial 30 minutes of adsorption in the dark highlights the high adsorption capacity of the synthesized Ag/Ag_2_O/γ-Fe_2_O_3_ NPs, which could attributed to their high surface area, available active sites and due to the electrostatic adsorption between the cationic dye and highly negative surface of the NPs.^83^ After the irradiation, the absorbance and peak intensity were reduced to near-complete removal of the dye for both types of irradiations (UVA and solar) under 60 minutes only, indicating the effective and fast photodegradation of crystal violet (CV).

Fig. 10(c and d) demonstrates the effect of initial dye concentration (10–70 mg L^−1^), and the non-linear kinetic fitting of PFO (*R*^2^ > 0.9) along with their apparent rate constants (*K*_app_) as an inset. In both light sources (UVA and Solar), *K*_app_ and the removal efficiency (*R*%) values decrease as the initial dye concentration increases. As the concentration increases from 10 to 70 mg L^−1^, under solar irradiation, *K*_app_ decreases from 0.12396 min^−1^ to 0.02162 min^−1^, and *R*% from 95.5% to 64.6%, while under UVA irradiation, *K*_app_ decreases from 0.15276 min^−1^ to 0.02417 min^−1^, and *R*% from a maximum degradation of 97.87% to 64.27%. These results suggest a saturation in the surface-active sites, because the sites and the adsorption capacity are finite at a constant photocatalyst concentration.^84^ Increased dye concentration leads to limited light penetration and absorption by the NPs, as fewer photons reach the surface lead to a reduction in the generation and availability of reactive oxygen species (ROS), which are needed for complete removal, as stated by previous studies.^84–86^ The Ag/Ag_2_O/γ-Fe_2_O_3_ NPs showcased a strong and fast photocatalytic oxidation at even 70 mg L^−1^, which is considered a moderate concentration. These results match previous studies on the photocatalytic degradation of CV, in which the reactions follow PFO and Langmuir–Hinshelwood kinetics.^84–86^ The difference between UVA and solar irradiation is negligible, confirming their efficiency under sunlight, which is attributed to their narrow bandgap energy (*E*_g_ = 1.934 eV) and their ability to absorb light at a wide range of wavelengths at both UV and visible regions. These results demonstrate a fast degradation with near-complete removal at lower concentrations in a short period of time of only 60 min of illumination, confirming the potential of the NPs for scale-up experiments and to be applied in water purification using an eco-friendly and economic method of synthesis design under sunlight irradiation.

The effect of the light sources alone (photolysis) was conducted and compared to photocatalysis at 30 mg L^−1^ to compare the effect of the presence of the photocatalyst, along with a dark adsorption experiment at the same concentration to assess the effect of the light source (Dark *vs.* illumination). The results shown in Fig. 10(f) indicate that photolysis (absence of Ag/Ag_2_O/γ-Fe_2_O_3_ NPs as a photocatalyst) was not efficient in the removal of CV dye, as presented with lower values in *R*% for both types of irradiation (20.16% degradation for UVA irradiation and 17.76% for solar irradiation), it showcases the importance of the Ag/Ag_2_O/γ-Fe_2_O_3_ NPs an their significant role in the removal of the dye. Meanwhile, dark adsorption has a removal efficiency of 75.09% at 60 min, although this result was achieved at about 30 min (*R*%_30 min_ = 73.49%), and it plateaus over time, indicating a saturation of the active sites on the surface of the green synthesized photocatalyst. This high but non-complete adsorption is significant for photocatalysis, as about 75% of pollutants are captured and adsorbed onto the surface of the NPs, facilitating the interfacial redox reactions for the further degradation of the dye using the generated ROS.

Scavenger tests were performed under UVA, 30 mg L^−1^ CV, and 0.5 g L^−1^ NPs to identify the main ROS (Fig. S2, SI) using *p*-benzoquinone (BQ; typically scavenges O_2_˙^−^/quenches e^−^), dimethyl sulfoxide (DMSO; ˙OH scavenger), isopropyl alcohol (IPA; commonly used as a ˙OH scavenger and could act as a sacrificial hole (h^+^) scavenger/electron donor), and disodium EDTA (EDTA; hole (h^+^) scavenger/electron donor).^87^ Compared with blank photocatalysis (92.47%), CV removal decreased strongly with BQ (30.18%) and DMSO (36.98%), while EDTA caused only a slight reduction (88.67%) and IPA gave a small enhancement (94.95%). Overall, the trends indicate that CV degradation proceeds mainly *via* O_2_˙^−^ and ˙OH, with a secondary contribution from h^+^ (see SI for details).

Comparison of the efficiency and reaction condition between green synthesized IONPs, and hybrid structures on in the photo/catalytic degradation of dyes, including Crystal Violet (CV) can be found in Table 1.

**Table 1:** Comparison of iron oxide and silver–iron based NPs in the photo/catalytic degradation of dyes

Plant name	Nanoparticle type	Type of degradation process	Experimental conditions	Degradation (%)	Reference
*Camellia sinensis* (leaf extract)	Amorphous iron oxide NPs (IONPs)	Photocatalytic degradation (sunlight)	Dye name: crystal violet	99.23	80
			NPs dose: 1 g L^−1^		
			Dye concentration: 10 mg L^−1^		
			Time: 210 min		
*Kulekhara* (leaf extract)	Ag–Fe_2_O_3_	Catalytic degradation (reduction using NaBH_4_)	Dye name: crystal violet and malachite green	100	29
			NPs dose: 0.5 mL of NPs solution		
			Dye concentration: 100 mg L^−1^		
			NaBH4 concentration: 0.5 g L^−1^		
			Time: 3 min		
*Palmyra* (sprout extract)	Ag@Fe bimetallic NPs	Photocatalytic degradation (sunlight)	Dye name: malachite green	91.23	35
			NPs dose: 0.05 g L^−1^		
			Dye concentration: 10 mg L^−1^		
			Time: 180 min		
*Cistus Monspeliensis* (leaf extract)	Ag/Ag_2_O/γ-Fe_2_O_3_ NPs	Photocatalytic degradation (UV light)	Dye name: crystal violet	97.87	This study
			NPs dose: 0.5 g L^−1^		
			Dye concentration: 10 mg L^−1^		
			Time: 60 min		
*Cistus Monspeliensis* (leaf extract)	Ag/Ag_2_O/γ-Fe_2_O_3_ NPs	Photocatalytic degradation (sunlight)	Dye name: crystal violet	95.50	This study
			NPs dose: 0.5 g L^−1^		
			Dye concentration: 10 mg L^−1^		
			Time: 60 min		

### Anticancer activity against human colorectal cancer (HCT-116)

The anticancer assay is presented as an initial proof-of-concept screening to assess the biological potential of the green-synthesized nanomaterial; detailed mechanistic investigations are beyond the scope of this study.

The *in vitro* cytotoxic effect was evaluated by the MTT assay method on the HCT-116 human colon carcinoma cell line treated with the green synthesized Ag/Ag_2_O/γ-Fe_2_O_3_ NPs, utilizing the *C. monspeliensis* aqueous leaf extract. The treated cell viability was compared with that of the untreated cells, clearly showing a concentration-dependent effect, which is consistent with the reported cytotoxic activity of NPs.^16,32,88^ Viability of cells was high at lower concentration levels ranging from 1 to 15.6 µg mL^−1^, with viability ranging from 63.96% to 99.76%, but was drastically reduced with moderate concentration levels, ranging from 41.87% to 18.45% for concentration levels of 31.25 to 125 µg mL^−1^, respectively. Higher concentration levels of 250 µg mL^−1^ and 500 µg mL^−1^ revealed high inhibitory percentage with the treated cells, exhibiting the strong dose-dependent cytotoxic effect with viability of only 9.03% and 3.17%, respectively.

**Fig. 11:**
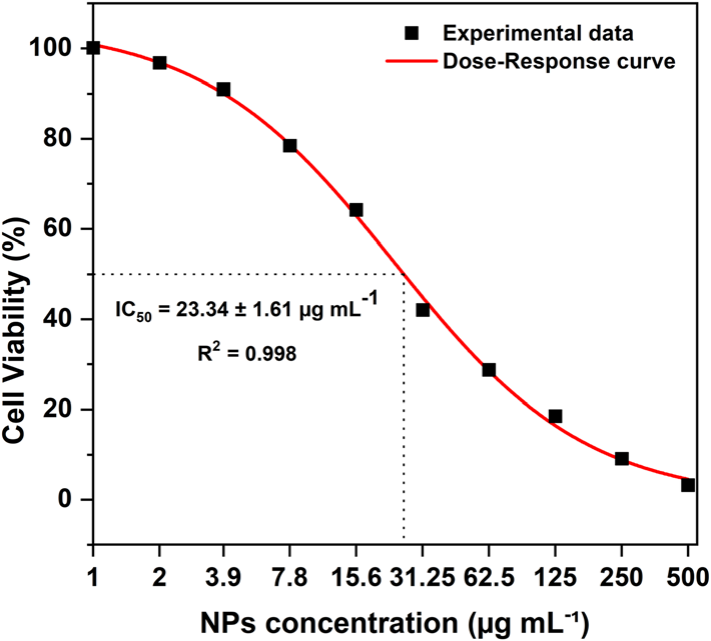
Relative cell viability of human colon carcinoma (HCT-116) cell lines treated with different concentrations of Ag/Ag_2_O/γ-Fe_2_O_3_ NPs with an estimated IC_50_ = 23.34 ± 1.61 µg mL^−1^ using the dose–response curve fitting (*R*^2^ = 0.998).

The IC_50_ value, which is the concentration required to reduce the viability of the HCT-116 cells to 50%, was calculated from fitting the dose–response curve (Fig. 11), it was found to be 23.34 ± 1.61 µg mL^−1^, indicating good anti-proliferative activity under the assay conditions, as lower IC_50_ values indicate greater biological activity.^46^

**Fig. 12:**
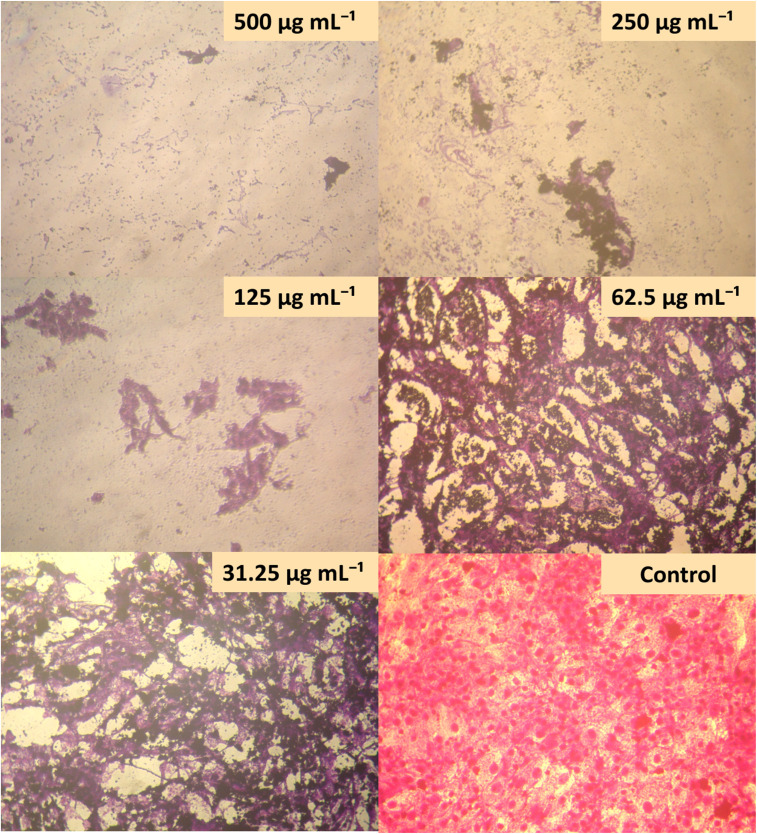
Morphologies of human colon carcinoma (HCT-116) cell lines as observed by optical microscopy after treatment with different concentrations of Ag/Ag_2_O/γ-Fe_2_O_3_ NPs.

Inverted optical morphological analysis supported the MTT assay data (Fig. 12). Untreated HCT-116 cells maintained their typical polygonal morphology with strong cell adhesion properties.^89^ Cells treated with 125–500 µg mL^−1^ of Ag/Ag_2_O/γ-Fe_2_O_3_ NPs revealed severe morphological alterations, including cell rounding, reduction and shrinkage, surface membrane dents, loss of adhesion, with reduced cell confluence, all of which are strong indicators of cell toxicity.^32,88^ Also, treatment with 31.25–62.5 µg mL^−1^ revealed cellular rounding with the initial stages of cell detachment, indicating the process of apoptosis.^88,89^

The possible mechanism underlying the observed anticancer activity of Ag/Ag_2_O/γ-Fe_2_O_3_ NPs is likely connected with the induction of oxidative stress, which is explained by the synergistic effect of the presence of iron along with silver, which leads to an increase in the production of reactive oxygen species (ROS) and an enhanced activity compared to IONPs alone.^90,91^ Silver NPs (AgNPs) are known to induce ROS production by disrupting the mitochondrial respiratory chain,^91^ while iron or iron oxides generate oxygen radicals through Fenton-like reactions.^92^

Silver NPs exhibit high cytotoxicity effect with IC_50_ values as low as 1.128 µg mL^−1^ against Triple Negative breast cancer cell line (MDA-MB-231),^93^ and 5 µg mL^−1^, against colorectal cancer cell lines HCT-116.^20^ Their activity was found to be dependent on the dose, size, shape, and surface functionalization of the NPs, as well on the type of the cells and synthesis method.^92,94^ However, the significant activity of AgNPs is non-selective, and can be toxic to even healthier cells, raising concerns about their therapeutic applications.^95^ IONPs, are recognized for their non-toxic properties, biocompatibility and biodegradability,^25^ they showcase cytotoxicity through many pathways including ferroptosis, oxidative stress, and apoptosis.^91,92^

Previous studies using IONPs have showcased an increase in oxidative stress indicators upon the treatment with NPs, HCT-116 cells treated with Gramin-loaded PVA-coated IONPs, with an IC_50_ of 25 µg mL^−1^ have elevated oxidative stress markers such as Nitric Oxide (NO), Lipid Peroxidation (LPO), and ROS levels,^88^ and a study on hepatocellular carcinoma (HepG2) cells treated with chitosan-coated Fe_3_O_4_ nanoparticles (IC_50_ = 39.15 ± 39.2 µg mL^−1^) reported elevated malondialdehyde (MDA) levels, a biomarker of lipid peroxidation (LPO), suggesting that the IONPs can induce oxidative stress.^22^ Although such formulations can exhibit good cytotoxic activity *in vitro*, some iron-oxide nanoparticle formulations have been approved for clinical use (*e.g.*, iron supplementation and/or imaging applications), supporting their overall biocompatibility and motivating their broad exploration in theranostics.^91^

The *C. monspeliensis* aqueous leaf extract used in the synthesis is expected to impart biofunctionality to the Ag/Ag_2_O/γ-Fe_2_O_3_ NPs surface and may further influence cellular uptake and reactivity,^38,96^ as the plant itself showed anti-inflammatory, analgesic activity, and antioxidant properties in earlier studies,^3,5,97^ that could enhance the therapeutic role of Ag/Ag_2_O/γ-Fe_2_O_3_ NPs.

The dual performance of Ag/Ag_2_O/γ-Fe_2_O_3_ NPs may be linked to their surface redox activity. In photocatalysis, scavenger tests indicate that ˙OH and O_2_˙^−^ are the dominant reactive species, reflecting efficient ROS generation under irradiation. In biological media, similar redox interactions and/or Ag-related effects may contribute to oxidative-stress-driven cytotoxicity; however, intracellular ROS measurements were not performed and should be explored in future work.

## Limitations and future work

Some advanced characterizations and follow-up photocatalytic tests were not available in our facility. Therefore, the nanoparticle architecture and elemental distribution could not be confirmed by HRTEM/EDX mapping, and oxidation states could not be verified by XPS. PL and Mott–Schottky analyses were also unavailable, so charge-carrier recombination and band parameters could not be evaluated. In photocatalysis, although spent-catalyst XRD showed no significant phase change, tests on common cations/anions (matrix effects) and byproduct identification were not performed. For the anticancer part, mechanistic assays such as intracellular ROS/apoptosis verification were not conducted. These points will be addressed in future work when instrument access becomes available.

## Conclusions

This study presents the successful and promising synthesis of Ag/Ag_2_O/γ-Fe_2_O_3_ nanoparticles prepared using a sustainable, plant-derived aqueous extract from *Cistus monspeliensis* leaves. Ag/Ag_2_O/γ-Fe_2_O_3_ NPs showed very high photocatalytic activities, giving excellent removal efficiencies of 97.87% under UVA irradiation and 95.50% under solar irradiation for Crystal Violet dye within a relatively short time of 60 minutes, hence proving their potential for environmental remediation purposes. Radical scavenger tests further confirmed that CV degradation proceeds mainly *via* O_2_˙^−^ and ˙OH, with a secondary contribution from h^+^ (SI). Moreover, the cytotoxicity observed against HCT-116 human colon carcinoma cells with an IC_50_ of 23.34 ± 1.61 µg mL^−1^ showed that these nanoparticles act not only as catalysts but also as potential therapeutic agents. Overall, the results support Ag/Ag_2_O/γ-Fe_2_O_3_ NPs as a green, multifunctional nanomaterial with strong photocatalytic performance and promising preliminary anticancer activity. Overall, this work establishes the green synthesis using *Cistus monspeliensis* as a novel biogenic source for Ag/Ag_2_O/γ-Fe_2_O_3_ NPs synthesis, and provides a foundation for developing multifunctional nanomaterials for environmental science, water treatment, and targeted cancer therapy.

## Conflicts of interest

There are no conflicts to declare.

## Data Availability

The data generated and/or analyzed during the current study are available from the corresponding author on reasonable request.

## References

[cit1] Bereksi M. S., Hassaïne H., Bekhechi C., Abdelouahid D. E. (2018). PJ.

[cit2] Nicoletti M., Toniolo C., Venditti A., Bruno M., Ben Jemia M. (2015). Nat. Prod. Res..

[cit3] Mechbal N., Bouhrim M., Bnouham M., Hammouti B., Karzazi Y., Kaya S., Serdaroğlu G. (2021). J. Mol. Liq..

[cit4] Sayah K., Chemlal L., Marmouzi I., El Jemli M., Cherrah Y., Faouzi M. E. A. (2017). South Afr. J. Bot..

[cit5] Al-Naqeb G., Zorzi G., Oldani A., Azzalin A., Avesani L., Guzzo F., Pascale A., De Giuseppe R., Cena H. (2024). IJMS.

[cit6] Ahmed S., Zengin G., Selvi S., Ak G., Cziáky Z., Jekő J., Rodrigues M. J., Custodio L., Venanzoni R., Flores G. A., Cusumano G., Angelini P. (2024). Pathogens.

[cit7] Amensour M., Pérez-Alvarez J. A., Skali-Senhaji N., Barnoussi N. E., Abrini J., Fernández-López J. (2024). AJPS.

[cit8] Haida S., Bakkouche K., Ouakki M., Galai M., Kribii A., Ebn Touhami M., Cherkaoui M., Kribii A. (2020). Mediterr. J. Chem..

[cit9] Zalegh I., Akssira M., Bourhia M., Mellouki F., Rhallabi N., Salamatullah A. M., Alkaltham M. S., Khalil Alyahya H., Mhand R. A. (2021). Plants.

[cit10] Habeeba U., Raghavendra N. (2024). Discov. Chem..

[cit11] Murtaza F., Akhter N., Qamar M. A., Yaqoob A., Chaudhary A. A., Patil B. R., Khan S. U.-D., Ibrahim N. A., Basher N. S., Aleissa M. S., Kanwal I., Imran M. (2024). Crystals.

[cit12] Sandupatla R., Dongamanti A., Koyyati R. (2021). Mater. Today: Proc..

[cit13] Endres T. H., Yimer A. A., Beyene T. T., Muleta G. G. (2025). Results Chem..

[cit14] S. A, Thamer A., R. K, P. A, R. V, M. K, Murad A., M. P (2020). J. Photochem. Photobiol., B.

[cit15] Selvaraj R., Pai S., Murugesan G., Pandey S., Bhole R., Gonsalves D., Varadavenkatesan T., Vinayagam R. (2021). Appl. Nanosci..

[cit16] Sreenivasa N., Meghashyama B. P., Pallavi S. S., Bidhayak C., Dattatraya A., Muthuraj R., Shashiraj K. N., Halaswamy H., Dhanyakumara S. B., Vaishnavi M. D. (2021). JEB.

[cit17] Alkahtane A. A., Alghamdi H. A., Aljasham A. T., Alkahtani S. (2022). Saudi J. Biol. Sci..

[cit18] Pillai R. R., Sreelekshmi P. B., Meera A. P., Thomas S. (2022). Mater. Today: Proc..

[cit19] Khan M. S., Alomari A., Tabrez S., Hassan I., Wahab R., Bhat S. A., Alafaleq N. O., Altwaijry N., Shaik G. M., Zaidi S. K., Nouh W., Alokail M. S., Ismael M. A. (2021). Pharmaceutics.

[cit20] Hamouda R. A., Hussein M. H., Abo-elmagd R. A., Bawazir S. S. (2019). Sci. Rep..

[cit21] Majeed S., Aripin F. H. B., Shoeb N. S. B., Danish M., Ibrahim M. N. M., Hashim R. (2019). Mater. Sci. Eng., C.

[cit22] Fahmy H. M., Shekewy S., Elhusseiny F. A., Elmekawy A. (2024). Cell Biochem. Biophys..

[cit23] Hmamouchi S., El Yacoubi A., El Idrissi B. C. (2022). Heliyon.

[cit24] Padilla-Cruz A. L., Garza-Cervantes J. A., Vasto-Anzaldo X. G., García-Rivas G., León-Buitimea A., Morones-Ramírez J. R. (2021). Sci. Rep..

[cit25] Endres T. H., Yimer A. A., Beyene T. T., Muleta G. G. (2025). Results Chem..

[cit26] Al-Zahrani F. a. M., Al-Zahrani N. A., Al-Ghamdi S. N., Lin L., Salem S. S., El-Shishtawy R. M. (2022). Biomass Convers. Biorefin..

[cit27] Jadhav V., Dhanwate Y., Raut P., Shinde S., Sawant R., Bhagare A. (2025). Discover Nano.

[cit28] Sarani M., Hamidian K., Barani M., Adeli-Sardou M., Khonakdar H. A. (2023). α-Fe_2_O_3_@Ag and Fe_3_O_4_@Ag Core-Shell Nanoparticles: Green Synthesis, Magnetic Properties and Cytotoxic Performance. ChemistryOpen.

[cit29] Kolya H., Kang C.-W. (2022). Sustainability.

[cit30] Habeeba U., Raghavendra N. (2024). Sustainable synthesize of beetroot extract-silver-iron oxide (BE-Ag-Fe_2_O_3_) bimetallic nanoparticles for antioxidant studies. Discov. Chem..

[cit31] Patel B., Choudhary N., Dudhagara D., Shahid M., Syed R., Yadav V. K., Sahoo D. K., Patel A. (2025). RSC Adv..

[cit32] Khan M. S., Alomari A., Tabrez S., Hassan I., Wahab R., Bhat S. A., Alafaleq N. O., Altwaijry N., Shaik G. M., Zaidi S. K., Nouh W., Alokail M. S., Ismael M. A. (2021). Pharmaceutics.

[cit33] Dharwadkar P., Zaki T. A., Murphy C. C. (2022). Hematol. Oncol. Clin. N. Am..

[cit34] Jadhav V., Dhanwate Y., Raut P., Shinde S., Sawant R., Bhagare A. (2025). Discover Nano.

[cit35] Sudhakar C., Selvam K., Poonkothai M., Ranjitha S. (2024). Inorg. Chem. Commun..

[cit36] Lazar M. M., Damaschin R. P., Volf I., Dinu M. V. (2024). Gels.

[cit37] Kulkarni S., Jadhav M., Raikar P., Barretto D. A., Vootla S. K., Raikar U. S. (2017). New J. Chem..

[cit38] Al-Zahrani F. A. M., Salem S. S., Al-Ghamdi H. A., Nhari L. M., Lin L., El-Shishtawy R. M. (2022). Bioengineering.

[cit39] Carroll D. O., Sleep B., Krol M., Boparai H., Kocur C. (2013). Adv. Water Resour..

[cit40] Laouini S. E., Bouafia A., Soldatov A. V., Algarni H., Tedjani M. L., Ali G. A. M., Barhoum A. (2021). Membranes.

[cit41] Fernanda L. V., Thais A. G., Caio C. A. R., Maria A. M., Ester R. G. (2015). Afr. J. Biotechnol..

[cit42] M. R, Uc J. R. J., Pinheiro D., Devi KR S. (2022). Appl. Surf. Sci. Adv..

[cit43] Mosmann T. (1983). J. Immunol. Methods.

[cit44] Taniguchi M., LaRocca C. A., Bernat J. D., Lindsey J. S. (2023). J. Nat. Prod..

[cit45] Haida S., Bakkouche K., Kribii A. R., Kribii A. (2021). Biochem. Res. Int..

[cit46] Murtaza F., Akhter N., Qamar M. A., Yaqoob A., Chaudhary A. A., Patil B. R., Khan S. U.-D., Ibrahim N. A., Basher N. S., Aleissa M. S., Kanwal I., Imran M. (2024). Crystals.

[cit47] Bouafia A., Laouini S. E., Tedjani M. L., Ali G. A., Barhoum A. (2022). Text. Res. J..

[cit48] Yogamoorthi A. (2015). Int. J. Nanosci. Nanotechnol..

[cit49] Niraimathee V. A., Subha V., Ravindran R. S. E., Renganathan S. (2016). IJESD.

[cit50] Alshehri A. A., Malik M. A., Patel R. (2021). J. Mater. Res. Technol..

[cit51] Gautam N., Singh K. B., Snigdha, Upadhyay D. D., Pandey G. (2023). RSC Adv..

[cit52] Sandupatla R., Dongamanti A., Koyyati R. (2021). Mater. Today: Proc..

[cit53] Kolya H., Kang C.-W. (2022). Sustainability.

[cit54] S S P., Rudayni H. A., Bepari A., Niazi S. K., Nayaka S. (2022). Saudi J. Biol. Sci..

[cit55] Dev M., Mukadam M., Bio W. J. (2025). Pharm. Health Sci..

[cit56] Pasinszki T., Krebsz M. (2020). Nanomaterials.

[cit57] Theivasanthi T., Alagar M. (2012). Electrolytic Synthesis and Characterizations of Silver Nanopowder. Nano Biomed. Eng..

[cit58] Dhoondia Z. H., Chakraborty H. (2012). Nanomater. Nanotechnol..

[cit59] Farooq N., Rehman A. U., Qureshi A. M., Rehman Z. U., Ahmad A., Aslam M. K., Javed H. M. A., Hussain S., Habila M. A., AlMasoud N., Alomar T. S. (2021). Surf. Interfaces.

[cit60] Purushotham D., Mavinakere Ramesh A., Shetty Thimmappa D., Kalegowda N., Hittanahallikoppal Gajendramurthy G., Kollur S. P., Mahadevamurthy M. (2025). IJMS.

[cit61] Gungure A. S., Jule L. T., Nagaprasad N., Ramaswamy K. (2024). Sci. Rep..

[cit62] Holder C. F., Schaak R. E. (2019). ACS Nano.

[cit63] Langa C., Mabuba N., Mahlaule-Glory L. M., Motaung D. E., Tetana Z., Hintsho-Mbita N. C. (2025). Int. J. Environ. Anal. Chem..

[cit64] Xue X., Gong X., Chen X., Chen B.-Y. (2021). J. Phys. Chem. Solids.

[cit65] Malik M. A., Alshehri A. A., Patel R. (2021). J. Mater. Res. Technol..

[cit66] Datye A. and DeLaRiva A., in Springer Handbooks, 2023, pp. 359–380

[cit67] Zagoskina N. V., Zubova M. Y., Nechaeva T. L., Kazantseva V. V., Goncharuk E. A., Katanskaya V. M., Baranova E. N., Aksenova M. A. (2023). IJMS.

[cit68] Biswal S. K., Panigrahi G. K., Sahoo S. K. (2020). Biophys. Chem..

[cit69] Sihem L., Hanine D., Faiza B. (2020). Nanotechnol. Russ..

[cit70] Vollath D. (2023). Nanoarchitectonics.

[cit71] Rahman I. A., Vejayakumaran P., Sipaut C. S., Ismail J., Chee C. K. (2008). Ceram. Int..

[cit72] Carlino E. , in Transmission Electron Microscopy Characterization of Nanomaterials, 2013, pp. 89–138

[cit73] Aritonang H. F., Koleangan H., Wuntu A. D. (2019). Int. J. Microbiol..

[cit74] Kumari M., Sadhu P., Talele C., Shah N. (2024). JNR.

[cit75] Habeeb Rahuman H. B., Dhandapani R., Narayanan S., Palanivel V., Paramasivam R., Subbarayalu R., Thangavelu S., Muthupandian S. (2022). IET Nanobiotechnol..

[cit76] Esparza R., García-Ruiz A. F., Velázquez Salazar J. J., Pérez R., José-Yacamán M. (2012). J. Nanopart. Res..

[cit77] Kulkarni N. S. (2019). Int. J. Pharm. Biol. Sci. Arch..

[cit78] Kampani A. (2024). Int. J. Adv. Biochem. Res..

[cit79] Aksu Demirezen D., Yılmaz Ş., Demirezen Yılmaz D., Yıldız Y. Ş. (2022). Int. J. Mater. Res..

[cit80] Yassin M. T., Al-Otibi F. O., Al-Askar A. A. (2023). Separations.

[cit81] Katubi K. M. M. , PhD thesis, University of Hull, 2015

[cit82] Loo Kiew P., Ainaa Mohd Fauzi N., Aufaa Firdiani S., Lam M. K., Tan L. S., Yeoh W. M. (2023). PROGEE.

[cit83] Jadhav A., Chavan R., Sonawane S., Kamble P., Mahajan S., Vhankhande B., Ghorpade R., Chougale A., Abd El-Salam N. M., Fouad H., Patil R. (2024). J. Nanoelectron. Optoelectron..

[cit84] Al-Asfar A., Zaheer Z., Aazam E. S. (2018). J. Photochem. Photobiol., B.

[cit85] Elashery S. E. A., Ibrahim I., Gomaa H., El-Bouraie M. M., Moneam I. A., Fekry S. S., Mohamed G. G. (2023). Magnetochemistry.

[cit86] Foroutan R., Peighambardoust S. J., Boffito D. C., Ramavandi B. (2022). Nanomaterials.

[cit87] Acharya S., Mansingh S., Parida K. M. (2017). Inorg. Chem. Front..

[cit88] Alnaim A. S. (2023). Ind. J. Pharm. Edu. Res..

[cit89] Hamida R. S., Albasher G., Bin-Meferij M. M. (2020). Cancers.

[cit90] Yang L.-X., Wu Y.-N., Wang P.-W., Huang K.-J., Su W.-C., Shieh D.-B. (2020). J. Mater. Chem. B.

[cit91] Singh P., Pandit S., Balusamy S. R., Madhusudanan M., Singh H., Amsath Haseef H. M., Mijakovic I. (2025). Adv. Healthcare Mater..

[cit92] Sidhic J., Aswathi M. K., Prasad A., Tom A., Mohan P., Sarbadhikary P., Narayanankutty A., George S., Abrahamse H., George B. P. (2025). J. Drug Delivery Sci. Technol..

[cit93] Morais M., Teixeira A. L., Dias F., Machado V., Medeiros R., Prior J. A. V. (2020). J. Med. Chem..

[cit94] Gomes H. I. O., Martins C. S. M., Prior J. A. V. (2021). Nanomaterials.

[cit95] Kovács D., Igaz N., Gopisetty M. K., Kiricsi M. (2022). IJMS.

[cit96] Al Sufyani N. M., Hussien N. A., Hawsawi Y. M. (2019). J. Nanomater..

[cit97] Ahmed S., Zengin G., Selvi S., Ak G., Cziáky Z., Jekő J., Rodrigues M. J., Custodio L., Venanzoni R., Flores G. A., Cusumano G., Angelini P. (2024). Pathogens.

